# Impact of regulatory measures on the approval timelines of advanced therapy medicinal products by the European Medicines Agency

**DOI:** 10.3389/fmed.2025.1623689

**Published:** 2025-06-26

**Authors:** Simonita Alaburde, Justinas Ivaska, Greta Kaspute, Tatjana Ivaskiene

**Affiliations:** ^1^State Research Institute Center for Innovative Medicine, Vilnius, Lithuania; ^2^Clinic of Ear, Nose, Throat and Eye Diseases, Faculty of Medicine, Institute of Clinical Medicine, Vilnius University, Vilnius, Lithuania

**Keywords:** gene therapy, somatic-cell therapy, tissue engineering, advanced therapy, marketing authorization, PRIME scheme

## Abstract

**Introduction:**

The study examines the impact of regulatory tools, including PRIority MEdicines (PRIME) scheme, on the marketing authorization (MA) timeline of advanced therapy medicinal products (ATMPs) approved by the European Medicines Agency (EMA).

**Methods:**

A retrospective analysis of EMA-approved ATMPs was conducted using publicly available European public assessment reports. Timelines from submission to approval, regulatory pathways, frequency of scientific advice (SA), and use of supportive mechanisms such as PRIME and Orphan designation were analyzed.

**Results:**

A total of 27 ATMPs were approved in the EU, 52% of which received PRIME designation and 74% held orphan status. PRIME participation was associated with a 42.7% reduction in time to MA (*p* = 0.001), and orphan designation with a 32.8% reduction (*p* = 0.021). PRIME-designated products also had fewer and shorter clock stops and more frequent scientific advice interactions.

**Discussion:**

The PRIME scheme facilitates earlier MA by supporting developers in addressing regulatory requirements more efficiently, shortening time to approval by approximately one year. The study underscores the value of early and frequent engagement with regulatory authorities and the need for tailored regulatory frameworks to support smoother approval processes. These insights can help developers better plan and optimize regulatory strategies. By demonstrating the measurable benefits of PRIME, this research supports its continued use to accelerate access for patients with high unmet medical needs.

## 1 Introduction

Advanced therapy medicinal products (ATMPs) represent a rapidly evolving class of biopharmaceuticals, including gene therapy medicinal products (GTMPs), somatic-cell therapy medicinal products (CTMPs), and tissue-engineered products (TEPs) (1). These therapies offer transformative treatment options for diseases that were previously considered untreatable, particularly in areas with significant unmet medical needs, such as rare genetic disorders, oncology, and regenerative medicine (2–16).

The European Union (EU) established a comprehensive regulatory framework for ATMPs under Regulation (EC) No 1394/2007, designed to support their development while ensuring safety, efficacy, and quality (1). This framework includes specific pathways such as conditional marketing authorization, approval under exceptional circumstances and accelerated assessment. In addition, developers may benefit from supportive designations such as orphan status and the PRIority MEdicines (PRIME) scheme. Introduced by the European Medicines Agency (EMA) in 2016, the PRIME scheme supports the development of medicines addressing unmet medical needs by enabling early and proactive regulatory engagement. PRIME offers several incentives to developers, including enhanced scientific advice (SA) procedures, early appointment of CHMP or Committee for Advanced Therapies (CAT) rapporteurs, and eligibility for accelerated assessment. Notably, small and medium-sized enterprises (SMEs) and academic applicants may receive free SA for the indication that qualified for PRIME designation. These applicants may also be granted Early Entry PRIME status based on proof of principle data. This provides access to a dedicated EMA product team and tailored guidance to help generate the evidence needed to secure full PRIME benefits (17). While these regulatory tools can reduce approval timelines, developers must still present comprehensive data to meet necessary standards, as assessed by EMA scientific committees including the CAT (18, 19). The post-approval phase remains critical to ensure continued safety and efficacy in the market (20).

Despite these initiatives, the development, evaluation, and MA of ATMPs remain complex and resource-intensive, requiring extensive regulatory engagement. While significant progress has been made toward approval of ATMPs in the EU, challenges persist in achieving timely market access, sustainable commercialization, and widespread clinical adoption (21–23). Factors such as long regulatory timelines, iterative review processes, and high development costs can hinder the efficient translation of these therapies from bench to bedside (24). Additionally, the withdrawal or non-renewal of MA for several ATMPs raises concerns about the long-term viability of these products in a competitive and highly regulated market (25).

In this study we analyzed the impact of existing regulatory measures on the timelines of ATMPs’ approvals in the EU, focusing on their regulatory pathways, timelines, and the role of supportive measures such as orphan designation and the PRIME scheme. While the EMA periodically publishes aggregated data on ATMP approvals and overall timelines, our study provides timeline comparisons across different ATMPs and estimates the direct effect on time to MA. We assess the effects of regulatory tools on specific milestones in the authorization process, including active evaluation time, clock stop duration and frequency. Our analysis could contribute to shaping regulatory practices and development strategies, offering support to both industry and academic communities.

## 2 Materials and methods

We performed a retrospective analysis of all currently EMA-approved ATMPs up to November 30, 2024. Data were extracted from publicly available European public assessment reports (26). The timings of marketing authorization approval (MAA) procedure milestones, orphan drug designation status, approval pathways, the use of PRIME scheme, and number of SA/protocol assistance (SA/PA) provided by EMA were recorded. The different MAA pathways were categorized as standard approval, conditional approval, and approval under exceptional circumstances. Start of the MA procedure was considered Day 1 and the EU approval date was determined by date when EC issued the MA. Glybera was excluded from the timelines analysis because it underwent two re-examination procedures, which significantly extended its time to MA and introduced regulatory steps not seen in other products. Including it would have skewed the analysis and compromised comparability across the dataset.

Statistical analysis was conducted using R v4.4.1 (27). In summary tables of continuous variables, data were presented as medians and first and third quartiles (IQR). In summary tables of categorical variables, data were presented as counts and percentages. Continuous variables were compared across two groups using the Wilcoxon’s Signed Rank test, and across more than two groups using the Kruskal-Wallis test. The time to MAA based on the number of PAs was estimated using linear regression.

We modeled the effect of PRIME scheme and the number of SA on D1 to MA using linear regression with D1 to MA as the outcome variable. We log-transformed D1 to MA because the data were left-skewed and log-normal. For the PRIME scheme model, we controlled for regulatory pathway and orphan status as confounding factors. For the number of SA model, we controlled for PRIME scheme using an interaction term. Model fit was ensured by examining linearity, the normality of residuals, homogeneity of variance and influential observations. All hypothesis testing was carried out at the 5% (2-sided) significance level.

## 3 Results

At the time of the analysis a total of 27 ATMPs were approved in the EU. These include 19 GTMPs (70%), 4 CTMPs (15%), 3 TEPs (11%) and 1 TEP, combined ATMP (4%). Among these, the MAs for Glybera and MACI were not renewed. Five additional products had their MAs withdrawn.

**FIGURE 1:**
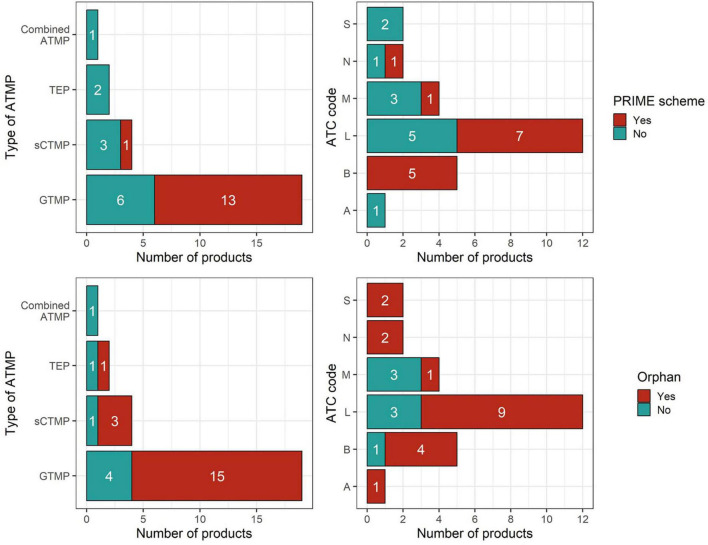
Distribution of authorized ATMPs by type and ATC first level, stratified by PRIME scheme eligibility (top) and orphan designation (bottom).

Of the approved ATMPs, 46% belong to the antineoplastic and immunomodulating agents’ group, as classified by the ATC first level. 74% of ATMPs hold orphan medicine status and 52% have obtained PRIME designation (Figure 1). Regarding the type of authorization, 52% of medicines were approved under standard conditions, 41% under conditional approval, and 7% under exceptional circumstances. Half of ATMPs started under accelerated assessment approval pathway, however, 69% of them were switched to standard during the course of the MA procedure.

### 3.1 Marketing authorization application timelines

**FIGURE 2:**
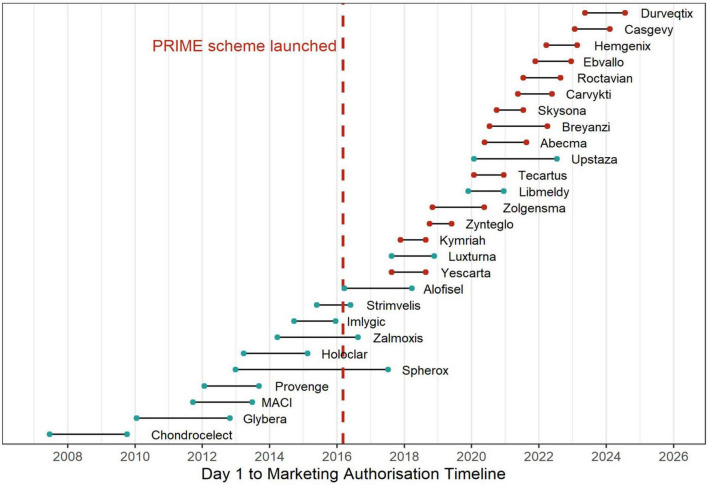
Timeline of ATMP approvals before and after PRIME scheme introduction.

Figure 2 illustrates the timeline from the start of MA procedure (“Day 1”) to MA for all EMA approved ATMPs between 2008 and 2024. The median time from the start of the MA procedure to final approval by the European Commission was 441 days (IQR 370–645, range 237–1656 days). Differences in median approval times were observed among ATMP types: 385 days for GTMPs (IQR 349–458, range 237–902), 660 days for CTMPs (IQR 539–766, range 386–876), and 1174 days for TEPs (IQR 933–1,415, range 692–1656). ATMPs with PRIME designation showed faster timelines, with a median of 376 days (IQR 324–426, range 237–627 days), compared to 669 days (IQR 459–848, range 364–1656 days) for those without PRIME designation. The total time from the start of the procedure to final approval was shortest for conditional approvals, with a median of 405 days (IQR 352–509, range 237–876 days), followed by standard approvals at 462 days (IQR 371–645, range 273–1656 days), and approvals under exceptional circumstances at 644 days (IQR 515–773, range 386–902 days) (Table 1).

**TABLE 1:** Summary of MA duration, clock stops, and scientific advice interactions.

Attribute	*N* = 26^1^	Day 1 to MA, days	Number of clock stops	Clock stops duration, days	Number of SA/PA
**Type of ATMP**					
Cell	4 (15%)	660 [539–766]	3.0 [2.8–5.0]	309 [220–389]	2.0 [1.0–3.5]
Gene	19 (73%)	385 [349–458]	2.0 [2.0–4.0]	125 [86–188]	4.0 [2.0–6.0]
Tissue	2 (7.7%)	1,174 [933–1,415]	2.5 [2.3–3.0]	889 [657–1,120]	3.0 [2.5–3.5]
Tissue, combined	1 (3.8%)	645 [645–645]	3.0 [3.0–3.0]	353 [353–353]	2.0 [2.0–2.0]
**Type of MA**					
Standard	13 (50%)	462 [371–645]	3.0 [2.0–4.0]	185 [100–353]	2.0 [1.8–4.3]
Conditional	11 (42%)	405 [352–509]	2.0 [2.0–5.0]	162 [87–210]	5.0 [3.3–5.8]
Exceptional circumstances	2 (7.7%)	644 [515–773]	2.5 [2.3–3.0]	342 [227–458]	2.0 [1.5–2.5]
**Orphan**					
Yes	19 (73%)	627 [519–742]	3.0 [3.0–4.0]	317 [211–402]	2.0 [1.3–2.0]
No	7 (27%)	385 [349–513]	2.0 [2.0–5.0]	111 [86–210]	4.0 [3.0–5.0]
**PRIME scheme**					
Yes	14 (54%)	376 [324–426]	2.0 [2.0–3.0]	99 [69–164]	4.5 [3.0–6.3]
No	12 (46%)	669 [459–848]	3.0 [2.8–5.0]	358 [178–455]	2.0 [1.0–4.3]
**Assessment Pathway**					
Accelerated	4 (15%)	353 [300–430]	2.0 [1.8–3.0]	108 [74–151]	4.0 [2.5–5.3]
Standard	13 (50%)	645 [448–838]	3.0 [3.0–5.0]	353 [165–450]	2.0 [1.0–5.0]
Switched to standard	9 (35%)	371 [333–405]	2.0 [2.0–3.0]	100 [64–162]	4.0 [3.5–6.0]

^1^n (%); Median [Q1–Q3].

The median time from Day 1 of the procedure to CHMP opinion was 386 days (IQR 309–582, range 204–1603 days). For ATMPs with PRIME designation, the timeline was shorter, with a median of 319 days (IQR 262–369, range 204–560 days), compared to 606 days (IQR 398–757, range 309–1603 days) for those without PRIME designation.

When excluding clock stops and considering only active evaluation time, the median time to CHMP opinion was 214 days (IQR 203–250, range 169–349 days). For ATMPs without PRIME designation, the median active evaluation time was 244 days (IQR 219–268, range 197–349 days), whereas for those with PRIME designation, it was 210 days (IQR 184–214, range 169–282 days).

Following the CHMP opinion, the EC issued a formal decision in a median of 60 days (IQR 55–63 range 33–102 days). For 8 ATMPs (27%) the approval decisions were made by consensus and for 19 (73%) by majority vote.

**FIGURE 3:**
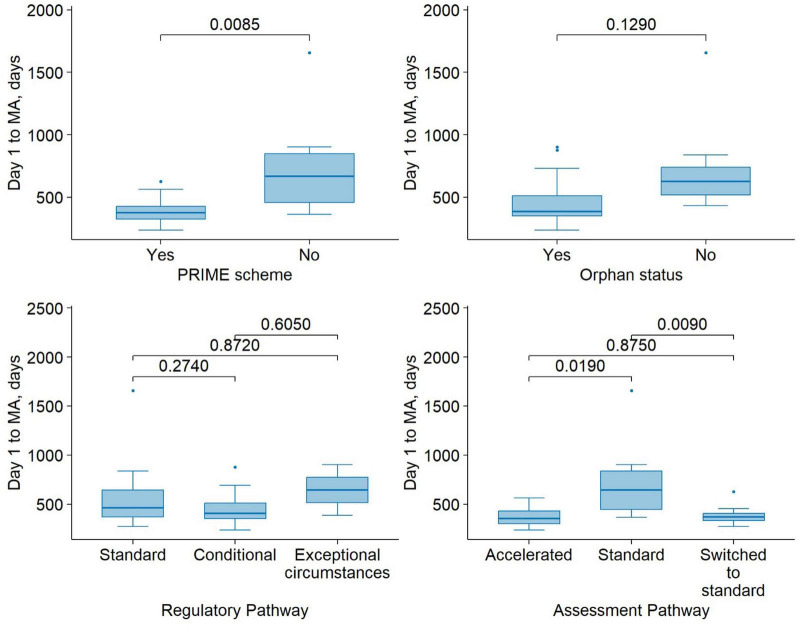
Time from submission to MA for medicinal products approved under different regulatory support mechanisms. Boxplots show the median (center line), first and third quartiles (box boundaries), and whiskers extending to 1.5 times the interquartile range (IQR). Points beyond the whiskers are shown as outliers. Pairwise comparisons were performed using Wilcoxon’s signed-rank test.

Using a linear regression model with D1 to MA on a log scale as the outcome variable, we examined the relationship between PRIME status and D1 to MA, controlling for regulatory pathway and orphan status. The presence of PRIME scheme on average decreased the duration of D1 to MA by 42.7%, the effect was statistically significant (β = −0.556, SE = 0.147, *p* = 0.001). The conditional regulatory pathway did not significantly differ from the standard pathway (β = 0.245, SE = 0.158, *p* = 0.137), nor did the exceptional MA condition (β = 0.417, SE = 0.255, *p* = 0.117). Having orphan status on average reduced the duration of D1 to MA by 32.8%, the difference was statistically significant (β = −0.398, SE = 0.16, *p* = 0.021). Figure 3 illustrates the distribution of durations from the start of the MA procedure (Day 1) to final approval, stratified by regulatory support measures and pathways.

### 3.2 Clock stops timelines

The median clock stop duration across all ATMPs was 164 days (IQR 92–344, range 26–1351). Clock stop timelines differed notably across product types, regulatory designations, and assessment pathways (Table 1). Gene therapies generally had shorter and fewer clock stops than TEPs, which showed the longest delays. Products with PRIME designation underwent fewer and shorter clock stops compared to non-PRIME products. Conditional approvals and accelerated assessment pathway were also associated with reduced clock stop duration and frequency. Notably, products initially assessed under the accelerated pathway but later switched to standard had clock stop characteristics similar to accelerated cases but experienced longer overall time to MA.

For ATMPs with PRIME designation, the first clock stop had a median duration of 58 days (IQR 35–95, range 26–125 days), and the second clock stop had a median duration of 32 (IQR 9–60, range 4–144 days), with durations decreasing progressively for subsequent clock stops. For ATMPs without PRIME designation, the first clock stop was longer, with a median duration of 169 days (IQR 117–270, range 87–1330 days), and the second clock stop had a median of 53 days (IQR 24–96, range 1–239 days), with durations decreasing progressively for subsequent clock stops. Notably, there were no fourth clock stops for medicines under the PRIME scheme. Overall, more than half (52%) of ATMPs underwent multiple rounds of clock stops related to outstanding issues.

### 3.3 Scientific advice and protocol assistance

All authorized ATMPs in the EU sought SA or PA from the EMA. The median number of consultations per product was 3.5 (IQR 2–5 range 1–8). Products with PRIME designation received more consultations, with a median of 4.5 (IQR 3–6.3, range 1–8), compared to a median of 2.0 (IQR 1–4.3, range 1–6) for those without PRIME designation. The majority of consultations addressed quality issues (92%), non-clinical concerns (88%), and clinical matters (96%). We used a linear regression model to estimate the effect of number of SA or PA on D1 to MA with PRIME scheme included as an interaction term. Among PRIME scheme ATMPs, each interaction reduced D1 to MA by 1.7% (β = −0.017, SE = 0.05, *p* = 0.731). However, this reduction is larger among non-PRIME scheme ATMPs and reduced D1 to MA by an additional 7.1% (β = −0.077, SE = 0.075, *p* = 0.318). The model did not find evidence in favor of a significant effect.

## 4 Discussion

The approval process for ATMPs in Europe is a highly regulated procedure, shaped by strict and comprehensive regulatory requirements (28). The EMA plays a pivotal role in this process, which is characterized by a centralized application system that facilitates the approval of ATMPs across all EU member states (29). However, the timeline for ATMP approval in Europe can be significantly longer than in other regions, such as the United States (30). Various factors influence ATMPs’ development timelines, including the selection of clinical endpoints for a specific indication, the structural properties of the medicine, patient recruitment challenges and number of treatment centers involved (31–33). The assessment of ATMPs appears to be a complex process, as suggested by the fact that only 27% of approval decisions were made by consensus. This lack of agreement may be due not only to scientific or regulatory challenges, but also to differences in ethical views, interpretation of limited data, risk tolerance, and opinions on how flexible the regulatory approach should be.

Our study aimed to evaluate the impact of existing regulatory measures on the actual approval timelines of ATMPs. Of all the regulatory tools evaluated, the PRIME scheme had the most impact on shortening time to MA. Our analysis showed that participation in PRIME scheme reduced the time to MA by 42.7% corresponding to approximately 1 year reduction. This highlights the scheme’s practical effectiveness in facilitating the evaluation of ATMPs and aligns with its intended goals to enhance regulatory support for promising medicines with high unmet medical need (17).

However, due to the diverse nature and complexity of ATMPs, approval timelines varied significantly, ranging from 8 months to 4 and a half years, while PRIME-designated products had a narrower range between 8 and 20 months. Zynteglo, a GTMP for transfusion-dependent β-thalassemia (TDT), had the shortest approval timeline (237 days). The swift approval in the EU was achieved by a combination of regulatory strategies. Zynteglo was granted PRIME status, enabling early and enhanced dialog between the EMA and the developer (34). The applicant received PA twice before the start of the pivotal trial (HGB-207). Recognizing its potential major public health benefit, the EMA conducted an accelerated assessment, shortening the standard review timeline. Given the unmet medical need in TDT and a favorable risk-benefit balance, Zynteglo received conditional MA. Notably, its first (and only) clock stop was the shortest among all ATMPs at just 26 days, with the CAT issuing their opinion 2 months after the applicant addressed the questions (35).

In contrast, Spherox, which is neither an orphan drug nor part of the PRIME scheme, followed the standard regulatory pathway, resulting in the longest approval timeline (1,656 days). Spherox was submitted for MA in 2013, 3 years before the PRIME scheme was introduced, meaning it could not have benefited from this regulatory support. The extended approval timeline was primarily due to a significantly prolonged first clock stop, lasting 1,351 days. Although EMA issued the final consolidated List of Questions to the applicant on April 25, 2013, the applicant did not submit responses until December 15, 2016 (36).

Medicines enrolled in the PRIME scheme may request accelerated assessment, which reduces the review period for MAAs by 60 days (37). A medicinal product qualifies for accelerated assessment if CHMP determines that it is of major public health interest, particularly from the perspective of therapeutic innovation. The concepts of unmet medical need and therapeutic innovation are key to this procedure, with unmet medical need defined as a condition for which there exists no satisfactory method of diagnosis, prevention, or treatment authorized in the European community, or a new medicinal product providing a major therapeutic advantage over existing treatments. For ATMPs in the PRIME scheme, applicants can receive confirmation during the clinical development phase that their medicine may qualify for accelerated assessment.

Our study found that while half of the ATMPs initially started under the accelerated assessment pathway, 69% of them were later switched to the standard pathway during the MA process. The EMA has acknowledged this trend as an area for future enhancements (33). The primary reasons for switching were quality issues or major clinical objections. These findings suggest that although accelerated assessment is an attractive pathway, many ATMP developers may not be sufficiently prepared to meet its requirements at the time of submission. Del Grosso et al. (38) analyzed the characteristics of drugs granted accelerated assessment and confirmed that it significantly reduces assessment time compared to the standard pathway. However, there is a high rejection rate for accelerated assessment (approximately 40%), suggesting a discrepancy between applicants’ perceptions of a product’s public health significance and the EMA’s evaluation criteria. This discrepancy underscores the need for clearer regulatory guidance or earlier scientific dialog to help sponsors better assess their eligibility and readiness for accelerated procedures. Our findings indicate that the median time to MA for accelerated assessment medicines was 12 months, while for those on the standard timeline, it was 22 months. Notably, switching to the standard timeline during the assessment did not significantly increase the total duration (median time 371 days). Clock-stop durations were slightly shorter in the group that was switched to the standard pathway compared to the accelerated assessment group (median of 100 vs. 108 days). However, this duration was notably shorter than in the standard pathway group (353 days), suggesting better preparedness among applicants.

Neither the use of conditional nor exceptional MA pathways showed statistically significant associations with D1 to MA duration. While the direction of the effects was positive, suggesting that non-standard pathways may be associated with longer timelines, the large standard errors and non-significant *p*-values indicate substantial variability and a lack of robust evidence for these associations in the current dataset. It is possible that other factors, such as the complexity of the data package, product type, or additional post-authorization commitments, may confound the relationship between regulatory pathways and timeline duration.

Orphan designation was significantly associated with shorter approval timelines, with an estimated 32.8% reduction in D1 to MA duration. This finding supports the potential time-saving effect of regulatory incentives provided to orphan-designated products. However, further investigation in larger datasets is warranted to explore whether this effect varies across product types or regulatory pathways.

Another key tool for expediting ATMP development is SA, which is part of the PRIME scheme and is provided by the EMA or national competent authorities. The primary purpose of SA is to help developers generate robust evidence regarding a medicine’s benefits and risks. It is particularly valuable for public bodies and small and medium-sized enterprises (SMEs), who may struggle to navigate regulatory requirements and ensure compliance throughout the development process. Most requests are initiated during the exploratory stage of development, suggesting that many developers seek advice early on, possibly due to inexperience (39). It has been shown that early regulatory dialog, before the pivotal trials, correlated with the positive MAA outcome (24, 40). Seeking comprehensive SA early on can prevent challenges during the design of pivotal studies. Resolving quality and non-clinical issues is essential to ensure smoother progress in clinical trials. Overlooking these aspects before starting the clinical phase can lead to unexpected issues and delays in pivotal trials and the approval process (39). As noted by Iglesias-Lopez et al., half of the approved products did not seek advice from the EMA before starting the main study. Our findings confirm that products in the PRIME scheme received more SA and PA, with a median of 4.5 consultations compared to 2.0 for non-PRIME products. This analysis explored the relationship between the number of PAs and the duration from D1 to MA, with a specific focus on whether this relationship differs between ATMPs with and without PRIME designation. The findings did not demonstrate a statistically significant effect of PA frequency on D1 to MA duration in either group. Among ATMPs with PRIME designation, each additional PA was associated with a modest and non-significant average reduction of approximately 1.71%. Interestingly, the interaction term suggests that the effect of PA may be more pronounced among non-PRIME products, with an additional average reduction of 7.1% per PA compared to PRIME products. However, this effect was also not statistically significant.

These results suggest that while PA is generally considered a key regulatory support tool, its impact on accelerating the regulatory timeline may be more nuanced. The lack of statistical significance may reflect heterogeneity in how PA is used, the complexity of individual development programs, or the possibility that the benefits of PA are more qualitative in nature - enhancing the quality of submissions rather than directly shortening timelines. Moreover, the smaller estimated impact of PA within the PRIME group could reflect a ceiling effect, where the overall regulatory acceleration provided by PRIME diminishes the marginal benefit of each additional protocol interaction.

Given the significantly shorter approval timelines and reduced clock-stop durations for PRIME products, it is evident that SA positively correlates with faster MA for ATMPs. This finding is consistent with the intent of the PRIME scheme to provide intensified guidance to facilitate the development of promising, innovative therapies (33).

The more pronounced distinction between PRIME and non-PRIME products emerged in the duration and frequency of clock stops used by applicants to address regulatory questions and issues. PRIME-designated products had shorter clock stops, with median durations of 58 and 32 days for the first and second stops, respectively. In contrast, non-PRIME products had longer clock stops, with median durations of 169 and 53 days for the first and second stops, as well as fewer clock stops overall. Shorter clock stops for PRIME products could be attributed to the amount and timing of SA used. For orphan products total clock stops duration was 317 and for products without orphan status - 111 days, reflecting the complexity of data requirements, manufacturing, and resource constraints that may attribute to longer response times during the MAA process. Future regulatory strategies might benefit from customized timelines based on product type and developer profile.

This study is subject to limitations, including the small sample size of approved ATMPs and reliance on publicly available data, which may not capture unpublished regulatory interactions. The lack of statistical significance may be attributed to the relatively small sample size, limiting the power to detect moderate effects.

## 5 Conclusion

The study confirms that the use of PRIME scheme shortens time to MA by approximately 1 year. Developers of ATMPs accepted onto the PRIME scheme were able to prepare their responses faster, resulting in earlier MA. Overall, the PRIME scheme appears to facilitate quicker access for patients to new medicines, supporting the timely evaluation of products that address unmet medical needs. The study draws attention to the importance of tailored regulatory frameworks. These findings provide valuable insights for manufacturers, as they offer a clearer expectation of the regulatory timeline and potential hurdles during the approval process. Furthermore, the results highlight the importance of engaging in early and frequent dialog with the EMA, as proactive regulatory interactions can significantly contribute to a smoother and more efficient approval process.

## Data Availability

The original contributions presented in this study are included in this article/supplementary material, further inquiries can be directed to the corresponding author.
